# Emerging Role of the Slit/Roundabout (Robo) Signaling Pathway in Glioma Pathogenesis and Potential Therapeutic Options

**DOI:** 10.3390/biom14101231

**Published:** 2024-09-29

**Authors:** Mariam Markouli, Athina Papachristou, Anastasios Politis, Efstathios Boviatsis, Christina Piperi

**Affiliations:** 1 Department of Biological Chemistry, School of Medicine, National and Kapodistrian University of Athens, 11527 Athens, Greeceathenapapachristou@gmail.com (A.P.); tsspolitis1@gmail.com (A.P.); 2 Department of Medicine, Boston Medical Center, Boston University School of Medicine, Boston, MA 02118, USA; 3 Second Department of Neurosurgery, “Attikon” University Hospital, National and Kapodistrian University of Athens, 15772 Athens, Greece; eboviats@med.uoa.gr

**Keywords:** Slit, Robo, axonal guidance, gliomas, angiogenesis, cell invasion, immune infiltration, immunotherapy

## Abstract

Gliomas represent the most common primary Central Nervous System (CNS) tumors, characterized by increased heterogeneity, dysregulated intracellular signaling, extremely invasive properties, and a dismal prognosis. They are generally resistant to existing therapies and only a few molecular targeting options are currently available. In search of signal transduction pathways with a potential impact in glioma growth and immunotherapy, the Slit guidance ligands (Slits) and their Roundabout (Robo) family of receptors have been revealed as key regulators of tumor cells and their microenvironment. Recent evidence indicates the implication of the Slit/Robo signaling pathway in inflammation, cell migration, angiogenesis, and immune cell infiltration of gliomas, suppressing or promoting the expression of pivotal proteins, such as cell adhesion molecules, matrix metalloproteinases, interleukins, angiogenic growth factors, and immune checkpoints. Herein, we discuss recent data on the significant implication of the Slit/Robo signaling pathway in glioma pathology along with the respective targeting options, including immunotherapy, monoclonal antibody therapy, and protein expression modifiers.

## 1. Introduction

Among the most common primary brain tumors [1,2], malignant gliomas and especially glioblastomas (GB; WHO grade 4 glioma) present highly aggressive tumor types, making up over 50% of glioma cases and signifying a dismal prognosis for patients [3]. GBs are invasive, angiogenic, proliferative, and molecularly diverse tumors that are generally challenging to cure because of their inoperative anatomical location and resistance to existing treatments [4]. Due to their poor prognosis, a lot of studies have investigated the pathogenesis of these tumors, aiming to detect novel molecular targets and develop new therapeutic strategies.

Baley and Cushing published the first classification of brain tumors in 1925 [5] and since then, the evolution of molecular biology has led to the elucidation of several signaling pathways and molecular defects that have a major impact in the pathogenesis of gliomas. The latest WHO Central Nervous System (CNS5) classification has therefore relied on the combination of histological with molecular parameters, employing novel molecular technologies (RNA expression profiling, Next-Generation Sequencing (NGS), and DNA methylation profiling) to update tumor grading, characterization, and prognosis [2]. CNS tumors are separated into six different families. The first one encompasses adult-type diffuse gliomas, which form most of the adult primary brain cancers, including isocitrate dehydrogenase (IDH)-wild type (WT) glioblastoma (GB). The remaining five families include pediatric low- and high-grade gliomas [a subgroup of which includes diffuse midline gliomas with histone 3 lysine 27 replaced by a methionine (H3K27M) mutation], confined astrocytic gliomas, glioneuronal and neuronal tumors, and ependymomas [2].

IDH-mutant diffuse gliomas now constitute the group of astrocytomas which are further subgrouped into different grades (2–4) with the use of Arabic numbers. The diagnosis of GB is based on the presence of IDH mutations, a crucial enzyme of the tricarboxylic acid or citric acid cycle [2]. The originally designated mIDH (IDH-mutated) GBs (now named IDH-mutant astrocytomas grade 4) and IDH-wild type GBs represent two independent, significant types of malignancies. IDH-wild type GB that represents roughly 90% of GB patients, is related to mutations at the promoter of telomerase reverse transcriptase (*TERT*), epidermal growth factor receptor (EGFR) amplification, and +7/−10 copy number alterations. On the other hand, mIDH grade 4 astrocytomas are associated with a homozygous deletion of cyclin-dependent kinase 2A/B (CDKN2A/B) [2,3].

Apart from specific genetic defects, several signaling pathways are altered in gliomas affecting a large range of molecular signaling cascades. These include the PI3K/AKT/PTEN, the TP53 and pRB, the JAK/STAT, the RAS/MAPK, the WNT, and the Hippo pathway, among others [6]. Additional implicated pathways involve angiogenic factors (VEGF, FGF, HIF1α, and Ang-1 and Ang-2), as well as metabolic regulators of glucose pathways, the citric acid cycle, glutamine, and lipids [3]. Mutations that cause a dysregulation in these pathways ultimately lead to cellular growth, the activation of anti-apoptotic genes, and tumor progression.

Research studies have now revealed the potential implication of Slit glycoproteins (Slit) and Roundabout receptors (Robo) in cell adhesion, angiogenesis, and metastasis along with the modulation of the tumor microenvironments of several tumor types, including gliomas [7]. Slits are secreted polypeptides that are evolutionarily conserved with variable physiological activities, exerting their function through binding to transmembrane Robo receptors [8,9]. Robos have a pivotal neurodevelopmental role in CNS, regulating neurogenesis, cell migration, and axon guidance in several anatomical areas, mostly in cortical and thalamocortical axons, but also in the spinal cord [9,10].

Slits have an amino-terminal fragment that directly binds and interacts with Robos in both physiologic and pathologic processes, such as angio- and organogenesis of the kidney, heart, lung, and mammary glands, but also inflammatory responses, tumorigenesis, and tumor vascularization. Upon Slit–Robo interaction, adaptor proteins are attracted to the cytoplasmic domain of Robos, inducing cytoskeletal modifications which in turn regulate cell adhesion, proliferation, and migration [10,11,12]. In this way, the Slit/Robo signaling pathway participates in the process of cellular communication of diverse systems (neuronal, renal, cardiovascular, etc.), regulating the guidance and polarization of neuronal and non-neuronal cellular types [7] along with cell motility, angiogenesis, and immune cell navigation [12]. In more detail, Slits/Robos are key players in kidney and heart development but also in the maintenance of normal organ structure and function. When dysregulated, this pathway can be involved in cystic kidney disease and renal cell carcinoma, as well as congenital heart defects, such as ventricular septal defects and Tetralogy of Fallot [10,11].

Similarly, given the contribution of the Slit/Robo pathway in CNS physiology and cellular homeostasis, it has been suggested that the aberrant expression or dysregulation of this pathway could have a significant primary role in related pathologies and brain tumor development [13]. Recent research has indeed indicated the implication of the Slit/Robo signaling pathway in glioma inflammation and immune cell infiltration, cell migration, and angiogenesis.

In this review, we used the keywords ‘Slit’ AND ‘Robo’ AND ‘gliomas’ OR ‘tumors’ OR ‘angiogenesis’ OR ‘treatment’ to retrieve research articles on PubMed that explore the structural and functional characteristics of the Slit and Robo protein family as well as current data implicating this signal transduction pathway in glioma pathogenesis. We also aimed to detect relevant targeting options that will allow us to exploit Slit/Robo signaling components in glioma therapeutics, including immunotherapy, monoclonal antibody therapy, and protein expression modifiers.

## 2. Structural Aspects of Slit and Robo Proteins

Three different Slit genes (*Slit1–3*) have been detected in vertebrates and only one in invertebrates [14,15]. The human chromosomes 10q24.1, 4p15.31, and 5q34-35.1 8 contain the *Slit1*, *Slit2*, and *Slit3* genes, respectively [8].

The Slit glycoproteins are composed of a group of single peptides with about 1500 amino acids, containing four leucine-rich repeats (LRRs: D1-D4), a laminin G-like module, nine EGF repeats, and a cysteine-rich knot [15,16]. Structural studies have shown that the LRRs of Slits are sufficient for Robo interaction while two Slit2 proteins form a homodimer through binding to each other via the fourth LRR domain [17].

**Figure 1:**
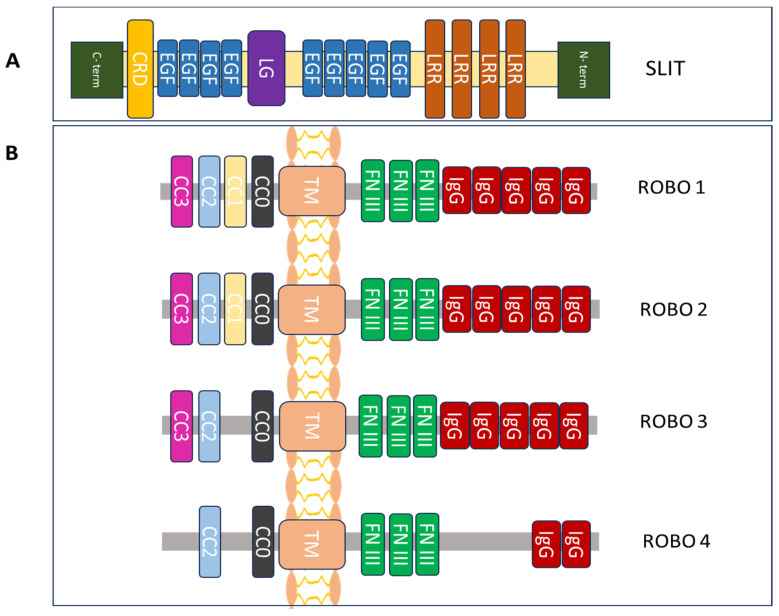
Structural components of the protein family Slit/Robo. (**A**) Slit glycoprotein structure expands from the C to the N terminus. It is made up of a cysteine-rich knot (CRD), nine epidermal growth factor (EGF) repeats, a laminin G-like (LG) module, and four leucine-rich repeats (LRRs). (**B**) The structure of the Robo family of proteins includes one conserved cytoplasmic domain (CCx), including CC0-3 (intracellular), one transmembrane (TM) region, three fibronectin type III (FN III) modules (extracellular) and five immunoglobulin (IgG) domains [14,22,23].

The human Slit proteins can be proteolytically processed to yield two fragments: the C-terminal (Slit-C) and the N-terminal (Slit-N) fragments. The four LRRs and five EGF repeats are present in Slit-N, while the remainder of the protein is found in Slit-C [18,19] (Figure 1). Extracellular secretion of either the whole protein or Slit fragments is possible [20,21], with different functional activities having been exhibited by each Slit fragment in vivo [22].

Four distinct Robo genes (*Robo1–4*) exist, originally detected in the nervous system of *Drosophila* in 1993 [23]. The study of Seeger et al. on the effect of *comm* and *Robo* mutation in the growth of CNS axon pathways in embryos of *Drosophila* detected that Robo gene mutations led to abnormalities in cell guidance during neurodevelopment [23]. The human 3p12.3 chromosome includes *Robo1* and *Robo2*, whereas *Robo3* and *Robo4* are found in the 11q24.2 chromosome.

Robos present a group of transmembrane receptor proteins that range from 1000 to 1600 amino acids in length [14]. They lack intrinsic or autocatalytic enzymatic activity and possess highly conserved intracellular regions. Robo proteins comprise one transmembrane region, followed by three extracellular fibronectin type III modules, five immunoglobulin-like (Ig-like) domains, and a conserved intracellular cytoplasmic domain (CC0, CC1, CC2, and CC3) [24]. Robo proteins that lack their basic enzymatic activity and autocatalysis in the intracellular space regulate downstream signaling by attracting other proteins or adaptors [14,15]. Of note, Robos are divided into many subtypes following transcription. Additionally, the variety and complexity of their function is mediated through interaction with other Robo receptors and the resultant loss of its extracellular domain following translation [15,16,17] (Figure 1).

The Slit–Robo interaction is achieved by the second Slit (D2) LRR domain, which is included in the Slit-N fragment and the first two N-terminal Ig domains of Robos [14]. The Slit D2 attaches itself to the Robo Ig1 domain via hydrophobic and electrostatic contact areas on its concave surface [25]. The disaccharide units of heparin sulfate (HS) are necessary to sustain Slit–Robo signaling [21]. In order to exert their cellular biological effects, Slits bind to their Robo receptors and trigger intracellular signaling pathways [22]. On the other hand, the Slit-C fragment cannot bind to Robo but it participates in the regulation of glucose metabolism, adipocyte thermoregulation, and neuronal axon guidance [21,23,26,27].

## 3. Regulators of the Slit/Robo Signaling Pathway

Several factors were detected to regulate Slit/Robo signaling, including activin A, GTPase-activating proteins, Abl tyrosine kinase, and Hox proteins. The study of Qin et al. showed that activin A and/or inhibin A modulate Slit and Robo mRNA expression during follicular development, depending on the diameter of the prehierarchical follicles. Upon activin A stimulation for 24 h, differential effects on Slit and Robo mRNA expression were observed in cells ranging from 4 to 8 mm in diameter [28].

Similarly, GTPase-activating proteins involved in neurite outgrowth and axon guidance can also modulate Slit/Robo expression. Chen et al. studied the impact of Slit/Robo GTPase proteins (srGAPs) on nerve regeneration after injury and detected three srGAP (1–3) family members expressed in mouse dorsal root ganglia (DRG) upon sciatic nerve transection (SNT) [29]. Following injury, increased mRNA and protein levels of srGAP1 and srGAP3 were observed in the ipsilateral DRGs compared to the contralateral L(3–4) DRGs from days 3 to 14 (peak days 7–14). The expression of SrGAP1 and srGAP3 was high in DRG neurons subpopulations in naïve DRGs. Of note, after peripheral nerve injury, DRG neurons with high srGAP3 staining were shown to co-express Robo2.

Additionally, a study of embryonic chick neural retinal cells showed that Slit/Robo signaling was associated with adhesion mediated through the N-cadherin and neurite outgrowth, in parallel with a Robo–N-cadherin cis-heteromultimeric complex formation [30]. The Abl tyrosine kinase that was linked with Robo was shown to induce N-cadherin inactivation by phosphorylating β-catenin [31]. The study of Rhee et al. [30] showed that upon Slit–Robo binding, the adaptor protein Cables moves towards the Robo-associated Abl and induces the formation of a multimeric complex through direct binding to β-catenin that associates with N-cadherin. This complex further induces the phosphorylation of β-catenin, N-cadherin loss of function, and nuclear transport of β-catenin.

Furthermore, Hox proteins are known to contribute to the guidance of axons during neural development. They can both act cell-autonomously within neurons and non-cell-autonomously in surrounding tissues to influence axon guidance decisions. The interactions between Hox proteins and Slits are complex and context-dependent [32]. Geisen et al. demonstrated that *Hoxa2* and *Hoxb2* genes regulate the migration of mouse pontine neurons during neural development via the Slit/Robo pathway [32] and they further showed that Robo2 represents a direct target of *Hoxa2*.

Apart of the previously mentioned proteins, several transcription factors have also been shown to control Slit expression in *Drosophila* embryos and mice. The longitudinal lacking (*lola*) gene is highly involved in the midline crossing of CNS axons during neuronal growth [33]. The Slit pathway prevents possible inappropriate actions during this period since embryos with non-mutated *lola* genes contain substantially lower levels (50%) of Slit proteins in the midline glia than *lola*-mutant embryos [33]. Additionally, it has been shown that CNS midline growth depends on other proteins, such as *Drosophila* single-minded (sim), which is a basic-helix–loop-helix (bHLH)-PAS transcription factor, Fish-hook protein, and insulin-related protein 2, which regulates the expression of *Slit* genes [34,35].

In terms of post-transcriptional regulation, the transmembrane protein commissureless (comm) composed of 370 amino acids but unknown domains has been shown to downregulate Robo during CNS development in *Drosophila* [36]. Robo was found to be highly expressed in ipsilateral and post-crossing commissural neurons, located in the plasma membrane, Golgi, and endoplasmic reticulum. Crossing commissural neurons expressed both comm and Robo, with Robo being expressed in low levels. On the other hand, Robo’s highest protein expression in these cells was present in lysosomes and late endosomes, where it was colocalized with comm, indicating that comm regulates Robo levels [37]. Moreover, during midline CNS development, Slit/Robo interaction was essential for the embryo, and potential defects had a health impact, including miscarriage [38]. Slit2, Robo1, and Robo2 expression plays a major role in this process, especially after crossing in mice spinal cords where their expression was reduced [39]. Rig1 or Robo3, a divergent member of the Robo subfamily, was highly expressed in commissural axons. Although its exact function is unknown, it seems that it coincides with Robo1 and modifies the latter in post-crossing neuronal cells [40].

Altogether, several factors have been shown to regulate the Slit/Robo pathway at both transcriptional and post-transcriptional levels during embryonic and neural development in *Drosophila* and mammals, with limited data in humans.

## 4. Physiological Roles of the Slit/Robo Pathway

Slit1 is predominantly expressed in the adult forebrain, but is also present in fetal brain, lung, and kidney tissues [41]. Slit2 is mainly expressed in the adult spinal cord, fetal lung, and kidney with lower levels detected in adult adrenal glands, thyroid and trachea [23,41]. Slit3 is mostly present in adult human thyroid cells [41]. Regarding the Robo family, Robo1 is found in most human tissues except kidney cells [42], Robo2 is expressed in neuronal and ovarian human cells [23], and Robo 3 is mainly present in ovarian cells [16] and Robo4 in endothelial cells, particularly of the ascending aorta [43].

The Slit/Robo axis has a major impact on the nervous system, being involved in axonal guidance and repulsion, as well as contributing to overall neocortex formation in mice [44]. In more detail, Slits can regulate cell differentiation, neuronal migration, axon guidance, and immune response. They transmit signals through Robo to physiologically guide neuronal cells during embryonic growth and are capable of stopping axons from traveling to the incorrect sites during nervous system development. A study by Kim et al. [45] demonstrated that neuronal cells are directed to their exit points in the spinal cord, following Slit signals, and spinal motor neurons were shown to express Robo1 and 2 during the early stages of cellular growth. Although Slits bind to Robo1/2 to mediate neuro-axon migration, several other ligands such as plexin A1, FLRT3, and NELL2 may be involved in neurocyte navigation through binding to Slits/Robos, including Robo3 [14].

Furthermore, it has been demonstrated that Slit proteins promote the division and elongation of cortical dendrites and sensory axons in vitro [14]. It was observed that when administered to adult rats and cultured DRGs, Slit1 enhanced neurite outgrowth and elongation. Being a significant nerve growth factor family member, Slit was shown to alter the cytoskeletons of neuronal cells by interrupting the expression of essential factors that play a role in the cellular phenotype, such as brain-derived neurotrophic factor and nerve growth factor [14]. The patterning of central and peripheral sensory neuronal branches can also be affected by the Slit/Robo pathway [14].

Furthermore, the Slit/Robo signaling pathway may interfere with blood–brain barrier (BBB) permeability. Slit2 is involved in the stabilization of BBB tight junctions via Robo4-mediated Rac1 activation. In turn, Robo4 upregulates Src and Erk1/2 phosphorylation by changing the expression of tight junction proteins, ZO-1, and occludin. Upon *Robo4* knockdown, an increased BBB permeability was observed due to reduced tight junction protein expression [46].

The Slit/Robo signaling axis may also regulate the polarity and motility of endothelial cells in controlling the formation of new blood vessels and angiogenesis [47]. Slit2 and Slit3 are produced by endothelial, perivascular, and vascular smooth muscle cells, while Robo1, Robo2, and Robo4 are expressed in endothelial cells of all types [48]. Studies in human umbilical vein endothelial cells (HUVECs) showed that Slit2 participates in cell migration by binding to Robo1 as well as in tube differentiation, causing increased formation of tubular networks in a dose-dependent manner. Neutralizing Slit’s function resulted in fewer and shorter tube structures, indicating that Slit2 offers a strong angiogenic activity in vitro [49]. Additionally, Slit3 has been shown to induce the migration of endothelial cells by binding to Robo4 through Rac1 and RhoA. Through modifications to endothelial cell motility and polarity, as well as interference with VEGF signaling, Slit/Robo signaling can inhibit or promote angiogenesis depending on the different target receptors [47]. In more detail, Robo4 can directly interact with paxillin and the ArfGAP (ADP-ribosylation factor-directed GTPase-activating protein), GIT1, to inhibit Arf6 activation in response to VEGF and fibronectin, thereby blocking VEGF/VEGFR signaling. Robo4 can also bind UNC5B, and inhibit pro-angiogenic signaling downstream of VEGF/VEGFR. On the other hand, Slit enhances angiogenesis by binding to a Robo1/Robo4 heterodimer, promoting endothelial migration, inducing the formation of filopodia, and interacting with cadherins [47].

Slit–Robo interaction also prevents leukocyte chemotaxis and may be used to prevent the infiltration of inflammatory cells. In more detail, Slit2 was shown to inhibit the activation of the cell migration mediators Cdc42 and Rac2, preventing human neutrophils from adhering firmly to the inflammatory vascular endothelial barrier [50]. Slit2 was also shown to reduce neutrophil recruitment to the site of inflammation [51,52,53]. Remarkably, it has been reported that Slit/Robo4 reduces the harmful response of the host to the pathogen-induced cytokine responses and fortifies the vascular barrier [54]. Another study showed the chemoattractant role of Slit-N for human neutrophils and the chemorepellent potential of the N-terminal Slit2 fragment (Slit2-S) [55].

Taken together, the physiological roles of the Slit/Robo pathway are quite diverse, ranging from predominant neuronal functions and the modulation of BBB permeability to the regulation of angiogenesis and immune cell infiltration.

## 5. Implication of the Slit/Robo Pathway in the Pathogenesis of Gliomas

A number of studies demonstrate that the Slit/Robo pathway is implicated in cancer onset and may exert both tumor-suppressing as well as tumor-promoting properties through the modulation of a series of cellular processes, such as cellular adhesion, cytoskeleton formation, chemotaxis, angiogenesis, lymphogenesis, and innervation. In the majority of tumors, Slit expression is either downregulated or absent, including gastric, lung, liver, esophageal, and breast cancer [56,57,58,59,60,61,62]. These findings are primarily related to promoter hypermethylation, suggesting that Slit/Robo pathway has an inhibitory effect on these cancers. On the other hand, some tumors, including melanoma and hepatocellular carcinoma and gastric and pancreatic cancer are characterized by an upregulation of Slit and Robo, suggesting that they may also act as oncogenes in some cancer types [60,61,62,63].

Regarding gliomas, initial studies have shown that Slit2 and Robo1 expression differs between healthy brain cells versus glioma cells. Slit2 expression is highest in control healthy brain cells and decreases in high-grade gliomas, and is therefore considered as a tumor suppressor. On the contrary, the percentage of Robo1-expressing glioma cells is higher compared to the control [64], with Robo1 being perceived as an oncogene.

However, a recent study showed that the expression of Slit2 increases with malignant development and correlates with immunosuppression and dismal patient survival [62]. The contribution of Slit2 to malignancy was attributed to the activation of PI3K-γ that further led to microglia/macrophage chemotaxis as well as tumor-supportive polarization. Furthermore, low Slit2 expression was associated with better prognosis as it inhibited macrophages, improved tumor vessel function and increased the sensitivity to chemotherapy and immunotherapy [7]. These data suggest that Slit 2 and Robo1 can be used as biomarkers in grading gliomas and predicting survival.

To this end, a recent study by Ozhan et al. employed the detection of specific Slit and Robo gene signatures (composed of *Robo1, 2, 3*, and *4* and *Slit1, 2*, and *3*) to calculate a univariable hazard ratio (HRuni) in order to predict cancer prognosis [65]. Their analysis revealed a significant differentiation between high- and low-risk prognosis patient groups based on specific Slit-Robo gene subsets, for low-grade gliomas that may prove useful in future personalized medical treatment. In more detail, Slit2 expression was associated with low-risk prognosis and low Robo3/high Slit3 were correlated with disease-free survival. Conversely, decreased Slit1 along with increased Robo2,4 expression was associated with poor prognosis in the low-grade glioma dataset.

In the following subsections, we discuss studies indicating the involvement of the Slit/Robo pathway in angiogenesis, tumor cell migration, and immune infiltration.

### 5.1. Slit/Robo’s Effects on Angiogenesis

The contributions of the Slit/Robo pathway’s effects in angiogenesis are mediated through Slit/Robo1 and Slit/Robo4. The secretion of Slit2 by tumor cells attracts Robo1 present in endothelial cells to migrate towards the tumor, inducing new blood vessel formation [66]. However, the interaction of Slit2 with Robo4 was shown to inhibit the migration of endothelial cells, exerting a negative impact in angiogenesis [67]. These studies indicate that Slit2 can have either a positive or a negative impact in angiogenesis depending on Robo1 or Robo4 binding. Moreover, overexpression of Robo4 has been observed in the placenta, brain, and bladder cancer as well as metastatic colorectal tumors to the liver [8,68].

**Figure 2:**
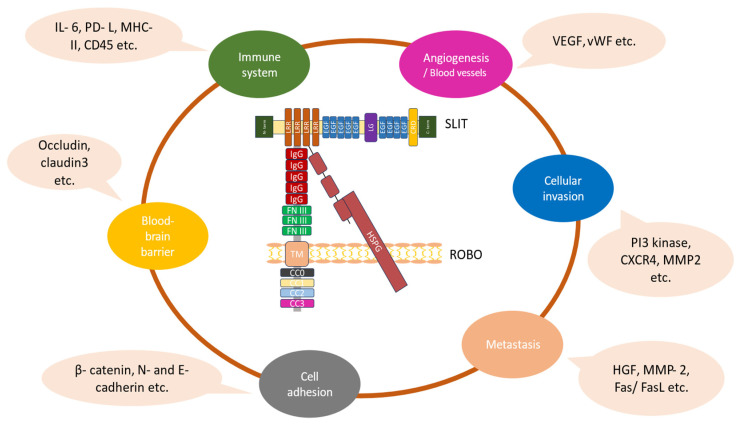
The diverse functional roles of the Slit/Robo pathway in gliomas. The Slit–Robo interaction is mediated by the second Slit (D2) LRR domain, which is included in the Slit-N fragment and the first two N-terminal Ig domains of Robos. The heparin sulfate proteoglycan (HSPG) disaccharide units are necessary to sustain Slit–Robo signaling. The binding of Slit proteins to Robo receptors mediates various cellular effects in gliomas associated with their growth and progression [22,23].

The angiogenic potential of Slit2 in human glioma cells was studied by implanting tumor cell spheroids via the cranial windows into tomato-fluorescence reporter mice. In control cells, the aberrant enlargement and loss of branching points was observed in the blood vasculature. In *Slit*-knockdown tumors, the blood vessels became less dilated and maintained their branches. The glioma arteries that overexpressed Slit2, on the other hand, were dilated and lost branchpoints sooner [7]. When compared to control tumors, better perfusion with less vascular leakage was shown in tumor vessels of *Slit*-knockdown cells. The latter study also showed reduced hypoxic areas that were positive for glucose transporter 1 immunostaining—(Glut1-positive)—in addition to improved vascular function as well as increased Glut1 coverage of blood vessels, partially indicating an improved BBB function. When comparing *Slit2*-knockdown to control tumors, a lower expression of the immunosuppressive PD-L1, PD-L2, and IL-6 was noted in tumor cells. Moreover, both Robo1 and Robo2 expression were detected in tumor cells, suggesting that Slit2 effects in glioma angiogenesis were mediated through these receptors (Figure 2) [7].

### 5.2. Slit/Robo’s Effects in Cell Migration and Invasion

Previous studies employing commercially available tumor cell lines have shown that Slit expression is downregulated in several tumors including gliomas, inhibiting cell migration and invasion (Figure 2) [40,41]. In highly invasive human glioma U87MG Ang2-expressing cells, Slit2 expression was involved in the inhibition of cell migration as well as tumor cell infiltration into the brain parenchyma in vivo [69]. It was further shown that Slit2-associated inhibition of glioma cell invasion was mediated through a blockade of Cdc42’s Rho GTPase activity, without any alterations in N-cadherin and β-catenin expression levels [69]. Similarly, Mertsch et al. [70] studied the role of Slit2 and Robo1 expression in glioma cell migration and showed that Slit2 was highly expressed in healthy neurons, and minimally expressed in astrocytomas and glioblastomas. Robo1 expression was mostly restricted to normal neurons, and overexpressed in astrocytic and glioblastoma cell lines. Furthermore, *Robo1* knockdown in glioma cells reversed the chemorepulsive effects of Slit2, confirming that Robo1 serves as the main Slit2 receptor.

Slit2 was also shown to inhibit medulloblastoma cell invasion in multiple models in vitro and this effect was rescued by the ectodomain of Robo1 [71]. Moreover, this study showed that although both glioma and medulloblastoma tumor cells express Slit2 and Robo1, the recombinant Slit2 protein inhibited only medulloblastoma invasion [64,71].

Following these original observations, recent studies employing murine GBM models [46] or patient-derived tumor spheres and xenograft models [43] demonstrate contradictory results, with the increased expression of Slit2 in gliomas being correlated with tumor progression and invasiveness [7]. Slit2 was found to act in different cells of glioblastoma tumors expressing Robo1 and Robo2. Upon *Slit2* knockdown, cell proliferation was not affected but cell migration was reduced in transwell chambers. Subsequently, spheroid invasion was decreased in fibrin gels along with the invasiveness of glioma shSLIT2 patient-derived cells implanted in nude mice in comparison to controls [7].

The Slit/Robo signaling axis exhibits also a significant impact in cancer metastasis. The study of Qin et al. showed that increased levels of Slit2 in the brain exert a chemoattractant function to breast cancer cells with decreased levels of Robo1. Of note, the PI3K/Akt/β-catenin pathway was closely associated with breast cancer progression. The inhibition of Slit2/Robo1 and therefore of the above-mentioned pathway leads to increased levels of MMP-9 in brain-selective breast cancer cells, suggesting the activity of the Slit2/Robo1 axis as an important parameter for brain metastasis in breast cancer patients, mediated via the PI3K/Akt/β-catenin/MMP-9 signaling pathway [72].

There is also evidence that Slit2N/Robo4 activates VEGF-C/VEGFR3, thus promoting lymphangiogenesis, with the lymphatic system being a common metastatic pathway for numerous tumors [73]. In hepatic cancer cells, increased Slit2 expression and Robo1 downregulation were shown to mediate enhanced cancer cell migration. Robo1 overexpression was further shown to elevate the expression of matrix metalloproteinases MMP-2 and MMP-9 as well as the membrane-type1 MMP (MT1-MMP), inducing metastasis [74]. Another study demonstrated that the inhibition of esophageal cancer cell metastasis was achieved through the inhibition of Cdc42 and FAK mediated by Slit2 activity [14].

In summary, it is evident that Slit2 activity is involved in cell migration and the invasion of glioma cells mediated primarily through Robo1.

### 5.3. Slit/Robo’s Effects on Immune Cell Infiltration

A study of high-grade gliomas assessed the TME changes that occur in murine GB models with dysfunctional Slit/Robo signaling. These changes may also most likely occur in humans with GB, as shown by the positive correlations between the expression of the studied genes in GB patient samples. Comparing Slit2-overexpressing tumors to Slit2-silenced tumors, infiltration of immune cells was decreased in the latter. Day 21 *Slit2*-knockdown tumors had fewer F4/80+ myeloid cells than day 18 Slit2-overexpressing tumors or day 21 controls, according to immunofluorescence examination of tumor sections. In *Slit2*-knockdown tumors, there was an increase in activated MHC-II+ Antigen-Presenting Cells (APCs) and a decrease in MRC1 (CD206)+ infiltrating immune cells. In control (CTRL) tumors, CD45+CD11b+F4/80+Ly6G–TAMs (tumor-associated macrophages) made up approximately 12% of all cells; in *Slit2*-knockdown tumors, this number was just 6%. In the shCTRL cases, less than 20% of TAMs were expressing MHCII and CD11c, with 80% exhibiting the pro-tumorigenic marker mannose receptor C-type 1 (MRC1). However, 50% of shSlit2 CT2A tumor TAMs produced MHC-II and CD11c and displayed a cytotoxic activation profile. Increased neutrophil and dendritic cell infiltration was also shown in shSlit2 tumors, but to a smaller number than TAMs [7,64].

In terms of molecular characteristics, TAMs from *Slit*-knockdown tumors showed a reduction in the expression of PD-L1 and PD-L2 inhibitors of T cell activation, increased expression of the cytotoxic genes *IL-1b*, *IL-12*, *TNF-α*, *Ccr7*, and *Cxcl10* and reduced expression of the tumor-supportive genes *TGF-β1*, *Mrc1*, *VEGF-a*, *Cd209a*, *MMP9*, *Arg1*, *Ccl19*, and *IL-10*. When comparing TAMs sorted from *Slit2*-knockdown tumors to controls, increased IFN-γ and lower IL-10 and VEGF-A protein levels were observed (Figure 2). In accordance with decreased VEGF-A expression, binding of soluble VEGFR1 (sFlt1) in vivo revealed that over 80% of CTRL and Slit2-overexpressing cells bound Flt1, whereas only approximately 40% of stromal cells in *Slit*-knockdown tumors bound sFlt1 [7,64].

Although TAMs were decreased in *Slit*-knockdown tumors, the overall number of tumor-associated lymphocytes (TALs) was raised by threefold, along with increased CD4+ and CD8+ T lymphocytes inside the tumor. Additionally, the Th1 response-related genes *Cxcl11*, *IFN-γ*, *IL-17a*, and *IL-2* were more expressed in CD4+ TALs of *Slit*-knockdown tumors compared to Th2 response-related genes, such as *PD-1*, *CTLA4*, *IL-10*, and *Cxcl10*. Additionally, CD8+ TALs in shSlit2 tumors displayed decreased levels of genes associated with CD8+ T cell fatigue (such as *Tim3* and *Lag3*) and elevated expression of the IFN-γ and granzyme B (GZMB) activation markers. Compared to controls, *Slit2*-knockdown tumors were also higher in infiltrating antitumor GZMB+ activated CD8+ T cells. These CD8+ TALs of *Slit*-knockdown cells were also higher in IFN-γ and lower in VEGF-A and IL-10 protein levels [7].

Slit2 was shown to induce dose-dependent chemotaxis of bone marrow-derived macrophages (BMDMs), isolated mouse microglial cells, and peritoneal macrophages (PMs). siRNAs were then used to silence Robo1 and Robo2 in macrophages to investigate whether Slit2 signaling via Robo receptors promotes macrophage migration, but this blocked Slit2-related macrophage migration. Production of a full-length rat Robo1 construct (Robo1FL) resistant to siRNA that includes the cytoplasmic signaling domain was shown to restore migration [7].

The phosphorylation of Akt, Erk1/2, and PLC-γ was observed in Slit2-treated microglial cells and BMDM. Tumor-promoting gene transcription alterations were also caused by Slit2 as seen by CEBPβ1 and Stat6 phosphorylation, which induce macrophage polarization towards a tumorigenic phenotype.

Slit2 treatment further induced gene expression associated with a tumor-promoting macrophage phenotype by activating MMP-9, VEGF-a, Mrc1, Ccl19, TGF-β1, IL-10, Cd209a, and Arg1. In contrast, genes related to a cytotoxic response, such as *IL-1β*, *Ccr7*, *Cxcl10*, and *TNF-α*, were not affected by Slit2 treatment but elevated by lipopolysaccharides (LPSs). In comparison with cells not treated with Slit2, microglia and macrophages treated with Slit2 exhibited higher amounts of IL-10 and VEGF-a in their conditioned media. Of note, changes in gene expression induced by Slit2 were reliant on Robo1 and Robo2, as demonstrated by *Robo1/2* silencing, which reversed all Slit2 effects on gene expression and protein phosphorylation [7].

## 6. Therapeutic Targeting of the Slit/Robo Pathway

There have been various attempts to target the Slit/Robo signaling pathway in the context of cancer treatment. Several studies using Robo monoclonal antibody (mAb) therapy have already resulted in beneficial outcomes to treat tumors, including hepatoma and non-small-cell lung cancer tumors [75]. Based on the observation that the GB microenvironment is less immunosuppressed after *Slit2* knockdown, it was hypothesized that these tumors would be more responsive to T cell-based immunotherapy. Anti-PD1 immune checkpoint inhibitors were therefore combined with *Slit2* knockdown, and this produced potent antitumor responses [7,23]. To explore whether Slit2 signaling is sufficient in macrophages to induce the stromal response on its own, mice with deletions in the macrophage Robo receptor were developed. Histological and MRI imaging analysis comparing *Robo*-knockdown tumors to controls showed a reduction in tumor size, as well as improved perfusion at 21 days after tumor implantation. In more detail, it was demonstrated that these tumors were characterized by a significant decrease in tumor-supportive MRC1+ cells, an increase in cytotoxic MHCII+ cells, a reduced total number of intratumor Iba1+ myeloid cells, and lower VEGF-A expression. In addition, T cell infiltration was higher in these tumors, demonstrating that the inhibition of the Slit/Robo signaling pathway in macrophages could shift the GB microenvironment towards a more cytotoxic, T cell-rich phenotype. This further revealed a reduction in the systemic immunosuppression after macrophage-specific knockout of *Robo1* and *Robo2*. Increased APC numbers circulating to lymph nodes, triggering antitumor T cell responses, were proposed to drive this effect [25].

Similarly, Robo1Fc, a Slit2 ligand trap protein, was administered to mice to achieve lower Slit2 levels, with vascular dysmorphia and tumor size being significantly reduced. A significant elevation of the cytotoxic MHCII+ cell levels was observed along with a reduction in tumor-promoting MRC1+ cells, with a shift to lymphocyte over neutrophil predominance in their blood stream, similarly to Robo receptor-knockdown mice. At 150 days post-implantation, 5% of the treated mice survived and this percentage increased to 45% when Robo1Fc was combined with temozolomide (TMZ). Remarkably, 80% of the treated mice survived for 90 days when Robo1Fc was combined with immunotherapy, resulting in enhanced antitumor responses.

As previously discussed, the Slit/Robo pathway has an important impact on the metastatic process. Bektur Aykanat et al. investigated the anti-neoplastic properties of the antioxidant compound silymarin in hepatocellular tumor cells and evaluated the expression of Slit2/Robo1 and CXCR4 protein levels by using immunoblotting and immunochemistry techniques. Low doses of silymarin decreased the above protein levels while high doses exhibited antitumor effects by increasing the level of Slit/Robo proteins [76].

It is widely known that cancer cells exhibit complex genomic alterations, such as somatic mutations, gene fusions, and copy number variations [77]. In particular, cervical cancers demonstrate hemizygous deletions in the chromosomal locations of *Slit* and *Robo* genes, which can be inactivated through the hypermethylation of their promoters. These alterations may help scientists to identify pre-cancerous lesions and/or develop a new therapeutic strategy, by targeting the epigenetic characteristics of each tumor [78].

Anti-angiogenic therapy is also a treatment choice for tumors, with most approved drugs targeting VEGF. Wang et al. used a monoclonal antibody against the Robo1 first IgG2b domain, to investigate the impact of Slit/Robo interaction in angiogenesis during oral tumorigenesis in human tissues of oral buccal mucosa. The increased expression of Slit2, Von Willebrand factor (vWF), and VEGF in neoplastic tissue was suppressed after mAb treatment along with angiogenesis in vivo [55]. A similar approach was also employed to reduce the effects of the Slit/Robo signaling pathway in tumor neovascularization [61].

Finally, the inhibitor of transforming growth factor beta (TGF-β), galunisertib, has been used to modify Robo1 and Robo2 expression in pancreatitis and pancreatic ductal adenocarcinoma (PDAC) mouse cells. Cell cultures from mice lacking epithelial Robo2 exhibited elevated activity of Robo1 myofibroblasts and induction of TGF-β and Wnt signaling pathways. Galunisertib suppressed these effects and has been proposed as a therapeutic inhibitor for PDAC [79].

## 7. Conclusions and Future Perspectives

Taken together, the Slit/Robo pathway is implicated in diverse functional processes in different cells and plays a critical role in organ development, cellular proliferation, motility, and migration, adhesion, angiogenesis, and apoptosis in both normal and tumor cells. In gliomas, the Slit/Robo pathway is primarily involved in cell migration and invasion, angiogenesis, and tumor growth, as well as in the crosstalk between glioma cells and their microenvironment, affecting tumor progression and response to therapy. In this context, there is no doubt that the Slit/Robo axis is ultimately linked to brain tumorigenesis and glioma progression.

There are, however, various complex phenotypes and diverse outcomes associated with Slit/Robo signaling because of the great selection of ligands, receptors, and molecules that are involved in different tissues that need further investigation. It is highly demanded to elucidate how Slit and Robo interactions enable tumor development and angiogenesis as well as the underlying molecular mechanisms that allows them to exert these effects. More specifically, the pathways interacting with Slit/Robo to affect the cytoskeleton, cellular migration and invasion, proliferation, angiogenesis, and apoptosis need to be further elucidated and additional basic research needs to be carried out in this direction.

In this context, targetable molecules have yet to be identified to efficiently manipulate the Slit/Robo pathway. Therapeutic strategies may involve targeting the expression or stability of Slit proteins to potentially inhibit glioma cell migration and invasion. For example, since *Slit2* knockdown was shown to decrease tumor cell migration, it would be helpful to optimize systemic Slit2 inhibition and translate it into clinical practice for patients with high Slit2 expression. Additionally, using antagonists or antibodies to block Robo receptors can prevent the pro-tumorigenic effects mediated by these receptors, and targeting downstream signaling pathways activated by Robo receptors can disrupt the processes essential for glioma progression.

Currently, there are no FDA-approved therapies specifically targeting the Slit/Robo pathway in gliomas. Ongoing research is focused on understanding the complex biology of this pathway in relation to tumor development and progression, so that novel therapeutic targets and treatment strategies can be developed.

## Abbreviations

Roundabout (Robo); Central Nervous System (CNS); Glioblastoma (GB); Next-Generation Sequencing (NGS); Isocitrate dehydrogenase (IDH); Wild type (WT); Histone 3 lysine 27 replaced by a methionine mutation (H3K27M); Telomerase reverse transcriptase (TERT); Epidermal growth factor receptor (EGFR); Cyclin-dependent kinase 2A/B (CDKN2A/B); Leucine-rich repeats (LRRs); Immunoglobulin (Ig); Cysteine-rich knot (CRD); Laminin G-like (LG); Transmembrane (TM); Fibronectin type III (FN III); Dorsal root ganglia (DRG); Sciatic nerve transection (SNT); Basic-helix–loop-helix (bHLH); Blood–brain barrier (BBB); Human umbilical vein endothelial cells (HUVECs); ADP-ribosylation factor-directed GTPase-activating protein (ArfGAP); Matrix metalloproteinase (MMP); Antigen-Presenting Cells (APCs); Control (CTRL); Tumor-associated macrophages (TAMs); Mannose receptor C-type 1 (MRC1); Tumor-associated lymphocytes (TALs); Granzyme B (GZMB); Bone marrow-derived macrophages (BMDMs); Peritoneal macrophages (PMs); Lipopolysaccharides (LPS); Monoclonal antibody (mAb); Transforming growth factor beta (TGF-β); Pancreatic ductal adenocarcinoma (PDAC).
